# Stereotactic radiosurgery for intracranial cavernous malformations of the deep-seated locations: systematic review and meta-analysis

**DOI:** 10.1007/s10143-024-02434-9

**Published:** 2024-04-24

**Authors:** Salem M. Tos, Georgios Mantziaris, Ahmed Shaaban, Jason P. Sheehan

**Affiliations:** Department of Neurological Surgery, University of Virginia, Charlottesville, VA USA

**Keywords:** Intracranial, Cavernous malformation, Cavernoma, SRS, Hemorrhage, Deep-seated, Adverse radiation effect

## Abstract

**Objective:**

To determine the outcomes of stereotactic radiosurgery (SRS) for deep-seated (brainstem, basal ganglia, thalamus, cerebellar peduncle) intracranial cavernous malformations (ICMs).

**Methods:**

A systematic review and meta-analysis was performed according to PRISMA and MOOSE guidelines. The main outcomes were comparing pre- and post-SRS hemorrhage rates, using the pooled risk ratios (RR) as the measure of effect. Additionally, the study assessed lesion volume changes and radiation-injury incidence.

**Results:**

Data of 850 patients across 14 studies were included in the meta-analysis. The pooled RR of all deep-seated ICMs show a decrease in hemorrhage rate after SRS compared to pre-SRS over the total follow-up period (RR =0.13), initial 2 years (RR =0.22), and after 2 years (RR =0.07). For 9 studies that reported hemorrhage rate of the brainstem only, the pooled RR shows a decrease of hemorrhage rate after SRS compared to pre-SRS over the total follow-up period (RR =0.13), initial 2 years (RR =0.19), and after 2 years (RR =0.07). Volumetric regression was achieved in 44.25% and stability in 56.1%. The pooled incidence of symptomatic and permanent radiation injury was 9% (95% CI, 7–11) and 3% (95% CI, 0–1.9%), respectively.

**Conclusion:**

SRS appears effective in reducing hemorrhage rates for deep-seated ICMs. The risk of symptomatic radiation injury is low. Given the high risk of surgical morbidity, SRS is a reasonable treatment option for patients with deep-seated ICMs with at least one prior hemorrhage.

**Supplementary Information:**

The online version contains supplementary material available at 10.1007/s10143-024-02434-9.

## Introduction

Intracranial cavernous malformations (ICMs) are slow-flow, benign vascular lesions with that usually present with hemorrhage, seizures, and focal neurologic deficits [9]. Their prevalence is estimated to range from 0.2% to 0.5%, [2] with the cumulative 5-year risk of repeat bleeding approximating 31% for brainstem ICMs and exceeding 30% in thalamic lesions [10, 26].

Due to the eloquence of the location, hemorrhage of deep-seated ICMs in the brainstem, basal ganglia, and thalamus can result in significant morbidity and mortality [9]. While resection is recommended for symptomatic ICMs that are easily accessible, [2] it often results in excessive perioperative morbidity for those ICMs that are deeper seated [13, 14]. In their systematic review, Poorthuis et al. found that the overall risk of death and nonfatal stroke in the surgical arm significantly exceeded the analogous natural risk (6% over 2.4% over 5 years) for non-hemorrhagic ICMs [24]. The Angioma Alliance Scientific Advisory Board underscored the significance of ICM location on neurosurgical treatment decisions; despite the ever evolving technical adjuncts, postoperative morbidity is 5-8%, and mortality approaches 2% [2]. Stereotactic radiosurgery (SRS) represents an alternative treatment modality, with prior studies showing significant reduction of hemorrhage rate and epileptic activity after treatment [5, 6]. This systematic review and meta-analysis focuses on SRS outcomes for ICMs located in often less accessible, deep-seated regions of the brain that are often associated with high surgical morbidity risk.

## Methods

### Search strategy

Following guidelines specified in the Preferred Reporting Items for Systematic Reviews and Meta-Analyses (PRISMA) and the Meta-Analysis of Observational Studies in Epidemiology (MOOSE), we systematically searched electronic databases, including MEDLINE, Scopus, Web of Science, and Embase, spanning from each database's inception to January 9, 2024. Our search strategy involved a combination of relevant keywords and standardized index terms. A detailed list of search terms and their combinations can be found in Supplementary Table 1.

### Selection criteria and quality assessment

#### Type of studies

Prospective and retrospective observational studies of 10 or more patients diagnosed with intracranial cavernous malformation of deeply located structures (basal ganglia, thalamus, brainstem and/or cerebellar peduncles) based on clinical or radiographic assessments.

#### Types of intervention

Stereotactic radiosurgery (SRS) for intracranial cavernous malformations (ICMs) administered in a single fraction. SRS was performed using various technologies, including the Gamma Knife (GK, Elekta AB, Stockholm, Sweden), linear accelerators (LINAC), Cyberknife (Accuray Inc., Sunnyvale, California, USA), Rotating Gamma System (RGS-RS), or proton therapy devices.

#### Types of outcome measures

Studies should report at least one of the primary outcomes of interest, which included the annual hemorrhage rate, lesional volume changes, or radiation-induced changes (RICs).

#### Exclusion criteria

Studies that involved non-human subjects, were not in English, had a sample size below 10, used overlapping data from the same institution(s), and had a median follow-up duration of less than 1 year were excluded. Inadequate reporting of the outcomes of interest, such as hemorrhage rates without specifying the exact number of hemorrhages and the total patient-years before and after SRS, were excluded. Furthermore, Studies that do not specify the outcomes for deeply located ICMs separately were excluded. Additionally, studies including only reviews, case reports, abstracts, letters to the editor, and editorials were also excluded.

#### Quality assessment

Quality assessment of observational studies was performed using the “Risk Of Bias in Non-Randomized Studies of Interventions” tool (ROBINS-I).

### Screening and data extraction process

The study selection process involved screening titles, abstracts, and full-text articles using the Covidence systematic review software (Veritas Health Innovation, Melbourne, Australia). Three independent authors (SMT, GM and AS) conducted these screenings according to inclusion and exclusion criteria, and any conflicts that arose were resolved through discussion and mutual consensus.

### Data collection and variables

We extracted relevant data from each article, including study characteristics, baseline patient and ICM features, SRS parameters, and outcomes. Study data included the author’s name, year of publication, country and institution, treatment period, and study design. Demographic data included the number of patients, number of treated ICM, sex, age, clinical features, prior management, and follow-up duration. ICM data included location, volume, and hemorrhagic lesions. SRS parameters included SRS modality, and dosimetric parameters (margin dose and isodose line). Finally, the outcomes included annual hemorrhagic rate, lesion volume change, and rate of symptomatic/asymptomatic RICs. The criteria for diagnosing hemorrhage in ICM include the identification of a new bleeding event observed on computed tomography or magnetic resonance imaging studies involving the vascular malformation area. This observation may occur irrespective of the presence or absence of new or aggravated neurologic signs or symptoms.

### Statistical analysis

The primary focus for evaluating hemorrhage rates centered on comparing the pre-SRS observational period to the post-SRS observational time. The calculation formula for the hemorrhage rate involved dividing the total hemorrhage events (excluding the first hemorrhagic event if it led to ICM diagnosis) in all patients by the total patient-years in the respective observational periods. The post-SRS observational time was divided into the first 2 years and the subsequent period after 2 years post-SRS for two reasons. Firstly, the efficacy of SRS tends to become apparent after a latency period of 2 years. Secondly, there is an additional concern that the observed effect might actually mirror the natural history of ICMs, particularly given the clustering of hemorrhages. The risk ratio (RR) of the hemorrhage rate was computed across distinct observational periods: pre-SRS vs total follow-up period post-SRS, pre-SRS versus the first 2 years post-SRS, pre-SRS versus after 2 years post-SRS, and finally, the first 2 years post-SRS versus after 2 years post-SRS. A risk ratio (RR) <1 meant that SRS provided a decrease in the annual hemorrhage rate. This analysis was carried out using R version 4.3.1 (R Core Team (2021). R: A language and environment for statistical computing. R Foundation for Statistical Computing, Vienna, Austria URL https://www.R-project.org/). To assess the heterogeneity of results among the included studies, we employed Cochrane’s Q-test and the I^2^ statistic (URL https://handbook-5-1.cochrane.org/). For outcomes without significant heterogeneity, we applied the common-effects (fixed-effect) model, while the random-effects model was used for outcomes with significant heterogeneity. Heterogeneity was evaluated through a visual inspection of forest plots and measured using the I^2^ and chi-square (χ^2^) tests. The χ^2^ test determined the presence of significant heterogeneity, and the I^2^ test quantified its magnitude. A significance level (α) below 0.1 and I^2^ exceeding 50% was considered indicative of significant heterogeneity. Publication bias was assessed through a funnel plot and confirmed by Egger’s test. A p-value < 0.05 was considered statistically significant.

## Results

### Study selection

**Fig. 1:**
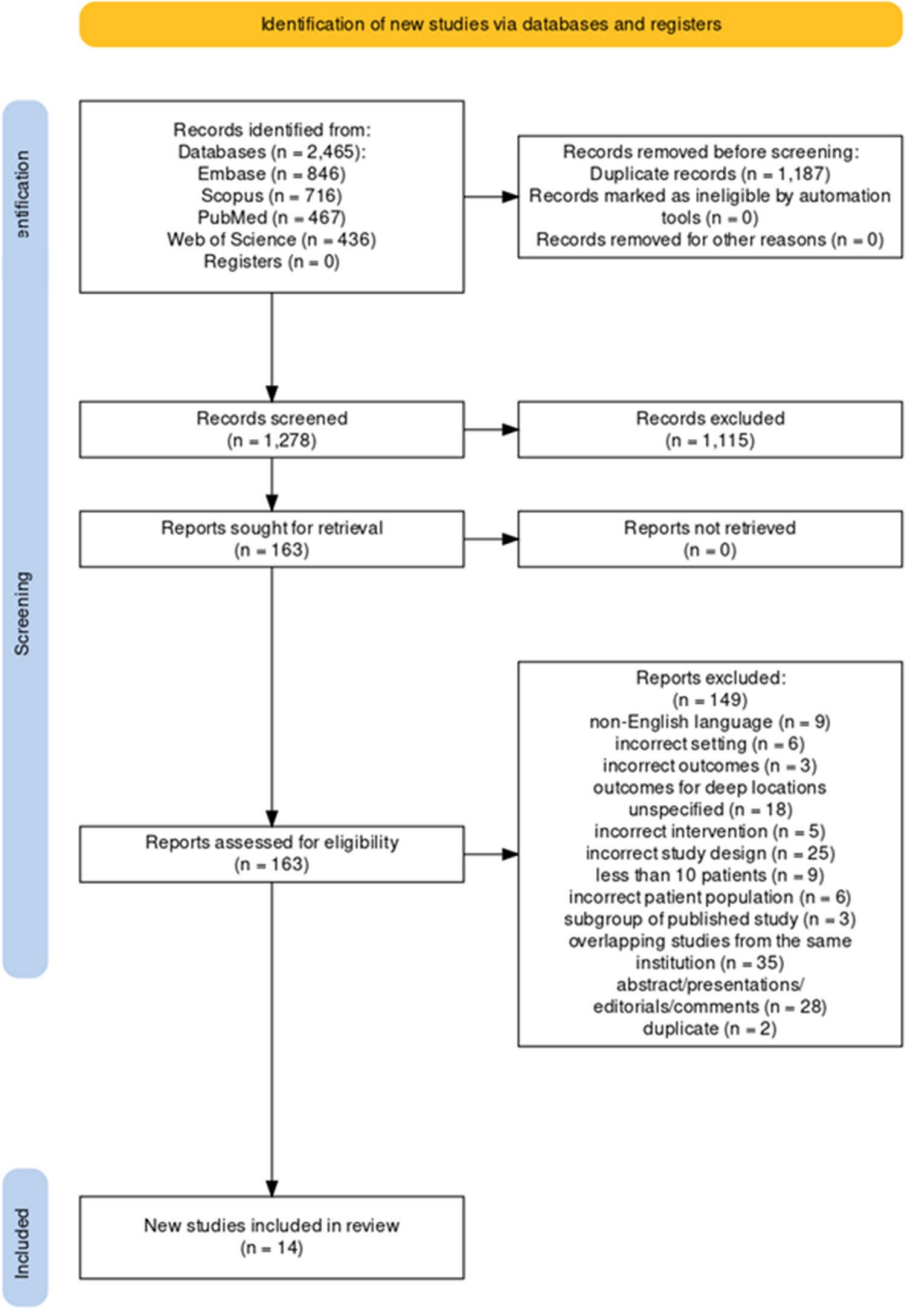
PRISMA flow chart of study selection process

The search resulted in a total of 2465 references, with 1179 records filtered for duplication using Covidence. Manual identification then uncovered an additional eight duplicates. After reviewing titles and abstracts, 163 citations were considered for full-text article examination. Of these, 149 studies were excluded for various reasons, including non-English language (*n* = 9), incorrect setting (*n* = 6), incorrect outcomes (*n* = 3), outcomes for deep locations unspecified (18), incorrect intervention (*n* = 5), incorrect study design (*n* = 25), less than 10 patients (*n* = 9), incorrect patient population (*n* = 6), subgroup of a published study (*n* = 3), overlapping studies from the same institution (*n* = 35), abstracts, presentations, editorials, comments (*n* = 28). Additionally, two duplicates were identified. Regarding overlapping data, when multiple publications originated from the same cohort, we included the dataset from the largest cohort. Finally, 14 studies eligible for at least one analysis were chosen in the current study [1, 7, 8, 11, 12, 15, 17, 19–23, 25, 27]. The updated PRISMA flow diagram (Fig. 1) illustrates the study selection process.

### Study, patient, and ICM characteristics

Out of the 14 studies incorporated into the analysis, 12 were retrospective cohorts, one was a case-control study, and one was a prospective clinical observation trial. Notably, 12 studies were conducted at single-center institutions, with the remaining two being multicenter studies. Patients were treated between 1987 and 2021 at a diverse array of institutions throughout the world. The analysis included a total of 850 patients with 855 ICMs who underwent single-fraction SRS. The mean age ranged from 36.6 to 43.1 years (median: 39.5 - 43.7). Among the 14 studies, 13 reported sex information for 640 patients, revealing that 324 (50.6%) were male. Of 855 deeply located ICMs, the brainstem was the most common location (67%), followed by basal ganglia and thalamus (31.2%), cerebellar peduncles (0.23%), and unspecified locations (1.5%). (Table 1)

**Table 1:** Baseline characteristics of 850 patients

Baseline characteristics	n (%)	Studies
Age (year)		14/14
Mean	36.6 - 43.1	
Median	39.5 - 43.7	
Sex		13/14
Male	324/640	
Female	316/640	
Location (anatomic)		14/14
Basal ganglia/thalamus	267/855 (31.2)	
Brainstem	573/855 (67)	
Cerebellar peduncles	2/855 (0.23)	
Unspecified (Thalamus/midbrain)	13/855 (1.5)	
At least one hemorrhage	798/812	13/14
Dosimetric parameter		
Target volume (cm3), mean (median)	0.282 - 3.2 (0.24 - 1.6)	14/14
Margin dose (Gy), mean (median)	11 - 14.8 (12 - 15)	14/14
Isodose line (%), mean (median)	50 - 62.14 (50)	12/14
Follow up (month), mean (median)	38.9 - 111.72 (32 - 121.9)	14/14

### SRS dosimetric parameters

The mean target volume varied from 0.282 to 3.2 cm^3^ (median range: 0.24 - 1.6 cm^3^). Among the different technologies used, the Gamma Knife was employed in 13 out of 14 studies and LINAC in 1 studies. No proton studies met the eligibility criteria. The mean margin dose ranged from 11 to 14.8 Gy (median range: 12 - 15 Gy) in a single fraction, and the mean isodose line ranged from 50 to 62.14% (median: 50%). The mean follow-up duration spanned from 38.9 to 111.72 months (median range: 32 - 121.9 months) from the time of SRS (Table 1).

### Annual hemorrhage rate

**Fig 2:**
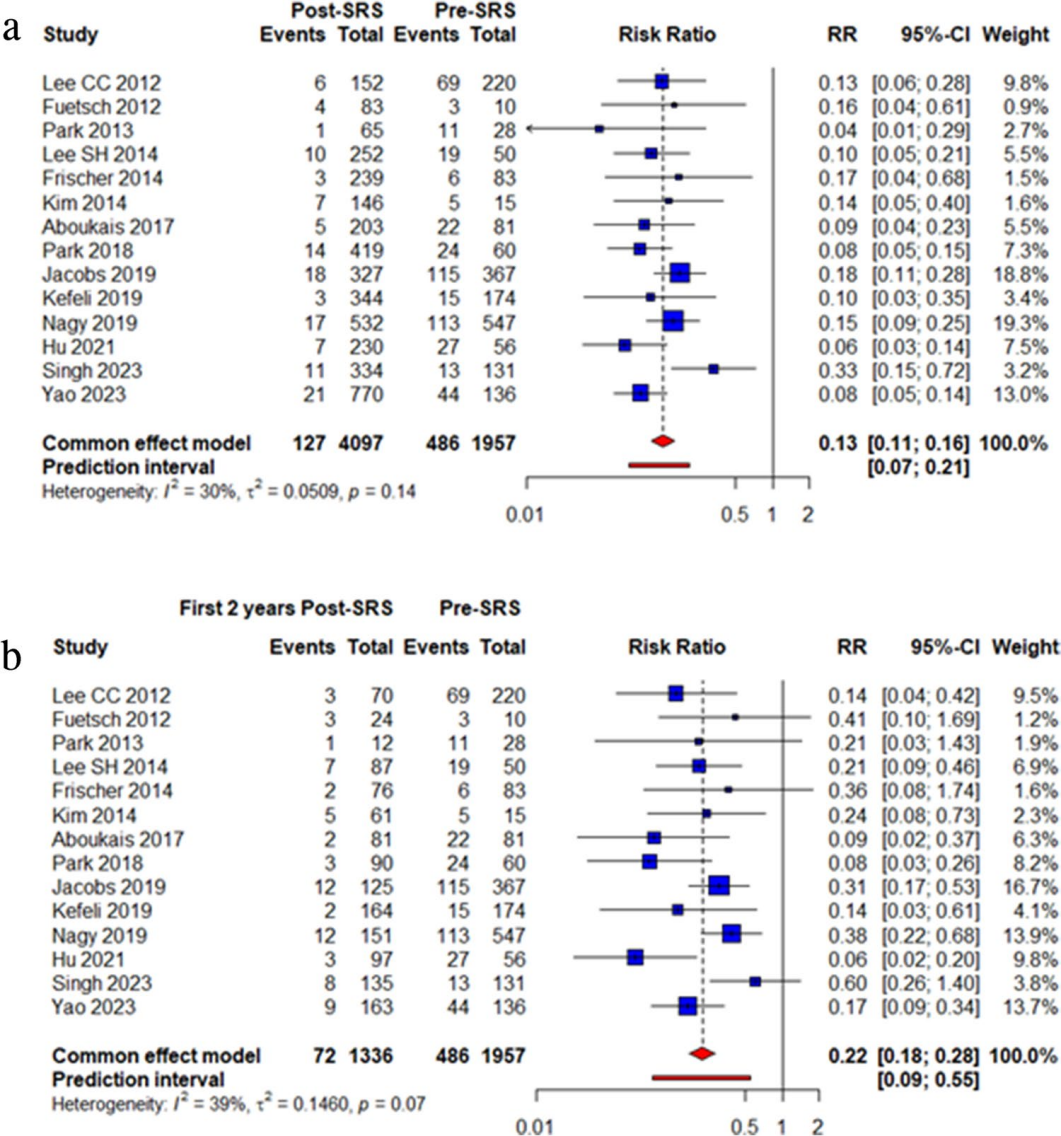
Forest plots of annual hemorrhage rate comparing pre-SRS and total post-SRS (**A**), pre-SRS and first 2 years post-SRS (**B**), pre-SRS and after 2 years post-SRS (**C**), and first 2 years post SRS and after 2 years post-SRS (**D**)

In comparing the pre-SRS annual hemorrhage rate to the total post-SRS follow-up period annual hemorrhage rate for deep-seated locations, the pooled risk ratio (RR) was 0.13 (95% confidence interval [CI], 0.11–0.16; *P* <0.0001, Fig. 2A). There was low heterogeneity among the included studies (*p*=0.14, I^2^ = 30%). In particular, when considering the 9 studies reported comparison of pre- SRS and total post-SRS annual hemorrhage rate of brainstem alone, the pooled RR was 0.13 (95% confidence interval [CI], 0.10–0.17; *p* <0.0001; heterogeneity, *p* =0.65, I^2^ = 0%, eFigure 2). The funnel plot revealed no apparent publication bias (eFigure 3), and Egger’s regression results indicated absence of statistically significant publication bias (t = -0.39, df = 12, p-value = 0.7051).

When comparing the pre-SRS hemorrhage rate to the first 2 years post-SRS rate for deep-seated locations, the pooled RR was 0.22 (95% CI, 0.18–0.28; *P* <0.0001, Fig. 2B). There was moderate heterogeneity among the included studies (*p*=0.03, I^2^ = 39%). In comparing the pre-SRS annual hemorrhage rate to the first 2 years post-SRS follow-up period annual hemorrhage rate for brainstem alone, the pooled RR was 0.19 (95% confidence interval [CI], 0.14–0.27; *P* <0.0001; heterogeneity, *p* =0.50, I^2^ = 0%, eFigure 4). Visual examination of the funnel plot suggested no potential publication bias (eFigure 5), and the results of Egger’s regression confirmed the absence of publication bias (t = -1.62, df = 12, p-value = 0.1304).

When comparing the pre-SRS hemorrhage rate to the post-SRS rate after 2 years for deep-seated locations, the pooled RR was 0.07 (95% CI, 0.05–0.09; *P* <0.0001, Fig. 2C). Notably, there was no significant heterogeneity among the included studies (*p*=0.99, I^2^ = 0%). In particular, when considering the pre- SRS and total post-SRS annual hemorrhage rate of brainstem alone, the pooled RR was 0.07 (95% confidence interval [CI], 0.05–0.10; *p* <0.0001; heterogeneity, *p* =0.96, I^2^ = 0%, eFigure 6). Visual examination of the funnel plot suggested absence of potential publication bias (eFigure 7), and the results of Egger’s regression confirmed the absence of publication bias (t = -0.12, df = 12, p-value = 0.9051).

Finally, there was a significant difference in the annual hemorrhage rate between the first 2 years post-SRS and after 2 years post-SRS rate for deep-seated locations (RR = 0.34; 95% CI, 0.24 – 0.48; *P* < 0.00001, Fig. 2D). Notably, there was no significant heterogeneity among the included studies (*p*=0.44, I^2^ = 0%). Furthermore, there is significant difference in the annual hemorrhage rate between the first 2 years post-SRS and after 2 years post-SRS rate of brainstem alone (RR = 0.37; 95% CI, 0.23 – 0.59; *P* < 0.00001; heterogeneity, *p* =0.35, I^2^ = 10%, eFigure 8). Visual examination of the funnel plot suggested no potential publication bias (eFigure 9), which was confirmed with Egger’s regression (t = 0.30, df = 12, p-value = 0.7706).

### Lesion volume changes and radiation-induced changes

Of ten studies assessing ICM volume changes, including 461 patients, 204 individuals demonstrated a reduction in lesion volume, accounting for 44.25% (Table 2). Stability in ICM volume was observed in 170 cases out of 303 patients across eight studies, yielding a pooled stable volume of 56.1%. Conversely, an increase in ICM volume (most studies reported as uncertain whether it resulted from true lesion progression or pseudo-progression due to radiation-induced changes or hemorrhage) was noted in 7 patients out of 303 across eight studies, constituting a rate of 2.3%.

**Table 2:** Lesion volume changes of the deep-seated intracranial cavernous malformations after SRS

Study	ICM volume change		
	Reduced (%)	Stable (%)	Increased (%)
Park 2013	10/22 (45.5)	12/22 (54.5)	0/22 (0) *
Kim 2014	24/39 (61.5)	13/39 (33.33)	2/39 (5.13)
Fuetsch 2012	3/12 (25)	9/12 (75%)	0/12 (0)
Park 2018	32/45 (71)	13/45 (28.9)	0/45 (0)
Kefeli 2019	49/82 (59.7)	NR	NR
Frischer 2014	18/ 38 (47.4)	20/38 (52.6)	0/38 (0)
Aboukais 2017	4/19 (21)	15/19 (79)	0/19 (0)
Jacobs 2019	32/76	NR	NR
Hu 2021	8/55	43/55	2/55
Singh 2023	24/73	45/73	3/73

* Computed as zero when all values are allocated to either reduced or stable categories; otherwise, indicated as NR (Not Reported)

The pooled proportion of patients who developed symptomatic RIC was 9% (95% CI, 7–11). There was no significant heterogeneity among the studies (*I*^2^ = 14%; *P* = 0.3; Publication bias, Egger test, *P* = 0.0011) (e10). Subgroup meta-analysis estimated the proportion of patients who developed symptomatic RIC as 8% (95% CI, 6–11; *I*^2^ = 33%) in the studies having a marginal dose of ≤13 Gy compared with 11% (95% CI, 7–15; *I*^2^ = 0%) in those having a marginal dose of >13 Gy (eFigure 11). The overall estimate of the proportion of patients who developed permanent AREs was 3% (95% CI, 0–1.9%). There was no significant heterogeneity among the studies (*I*^2^ = 0%; *P* = 0.97; publication bias, Egger test, *P* = 0.0007) (eFigure 12). The predominant imaging finding was perilesional edema, present in 7.2% (30 out of 418 patients) across ten studies. One case of cyst formation out of 549 patients (0.2%) was reported in 9 out of 14 studies. (eTable 2)

## Discussion

### Hemorrhage rate

While incidental ICMs are usually managed with active surveillance, [2] the cumulative risk of hemorrhage is not negligible, especially considering the young median age at presentation [3]. Additionally, patients that are symptomatic at presentation have reportedly a more aggressive disease course, experiencing higher rates of hemorrhage [5]. Deep-seated ICMs are associated with increased rates of hemorrhage and neurologic deficits [10, 26]. The annual repeat hemorrhage rate for thalamic ICMs has been reported to be as high as 9.7%, [26] while the 5-year estimated risk of intracerebral hemorrhage for symptomatic brainstem ICMs was 30.8% [10].

Given this aggressive course, it is generally recommended that symptomatic, deep-seated ICMs are treated [2]. Resection remains the mainstay treatment for select patients; complete resection can be achieved in the majority of patients that are surgical candidates [13, 14]. However, while potentially curative, resection often carries unacceptable perioperative morbidity and mortality for deeply-located lesions [2]. Two metanalyses focusing on outcomes following resection of ICM in the brainstem and basal ganglia/thalamus reported a pooled perioperative morbidity of 34.8% and 24%, respectively [13, 14]. SRS is an alternative, minimally invasive option for symptomatic ICM in these locations and can reduce the risk of hemorrhage [25].

Unlike arteriovenous malformations, ICMs are angiographically occult and thus radiographical obliteration is difficult to determine. While ICM volumetric decrease can be used as a surrogate outcome, it is rather inconsistent. As such, comparing pre- and post-SRS hemorrhage rates has been used to ascertain treatment efficacy. This meta-analysis that included 850 patients with 855 deep-seated ICM indicate that SRS afforded a significant decrease in the overall risk of hemorrhage following SRS compared to pre-SRS. This risk reduction was significant in the overall (RR=0.13, *P* <0.0001), first two years (RR=0.22, *P* <0.0001), and after two-year (RR=0.07, *P* <0.0001) periods. For studies reported hemorrhage rate of the brainstem only, the pooled RR shows a decrease of hemorrhage rate after SRS compared to pre-SRS over the total follow-up period (RR =0.13, *P* <0.0001), initial 2 years (RR =0.19, *P* <0.0001), and after 2 years (RR =0.07, *P* <0.0001). Similar to our results, a prior meta-analysis of 14 radiosurgical studies encompassing 576 patients with brainstem ICM noted a significant decrease of the post-SRS hemorrhage rate [16].

Given the absence of concrete radiographic evidence of SRS efficacy, there are concerns regarding the overlap between the natural history of untreated ICMs and the effect of radiation. An initial high re-hemorrhage rate (first 2-3 years after initial hemorrhage), namely the concept of temporal clustering, has been described; it has been suggested the difference in hemorrhage rates between the first two and later post-SRS periods can be attributed to this phenomenon [4]. However, Lee et al. demonstrated no significant difference when comparing the hemorrhage rates in the first 2 years after SRS in patients treated after multiple hemorrhages to patients treated after a single event [20].

### Adverse radiation effects and optimal planning

In this analysis the pooled incidence of symptomatic RIC at last follow-up was 9%; permanent symptomatic injury occurred at a pooled incidence of 3%. The predominant imaging finding was perilesional edema, present in 7.2% (30 out of 418 patients) across ten studies. This incidence of symptomatic RIC is higher compared to a recent report by Dumot et al.; in this multicenter study 5% of patients experienced neurologic deficits from radiation injury [5]. This difference is justified given the inclusion of patients treated for ICMs located in non-eloquent regions. In general, relatively high rates of symptomatic RIC have been reported for ICM, despite the relatively low prescription dose employed and the small target volume. It has been suggested that the hemosiderin ring might act as a radiosensitizer, leading to recommendations against its inclusion in the treatment volume [5]. The incidence of delayed adverse radiation events, such as cyst formation and radiation necrosis is much lower; Dumot et al. reported 2 cases of cyst formation, with one requiring stereotactic aspiration [5]. Radiation-induced ICM formation has also been reported but is rare; Koester et al. reported 10 cases after retrospectively reviewing a single-center database of 1662 treated with radiation treatment in the neck and head region [18]. Given the legitimate concern that radiation exposure may increase the formation of new lesions in patients with familial ICM, the Angioma Alliance recommends against utilizing SRS in the management of these patients [2].

Most studies generally recommend against exceeding the range of 11-13Gy prescription dose range in a single fraction to reduce the risk of adverse radiation effects. In the meta-analysis by Kim et al. on brainstem ICM treated with SRS, a significantly lower rate of AREs was observed with mean prescription doses ≤ 13 Gy [16]. These results were not replicated in our analysis; no significant higher incidence of symptomatic RIC was observed in studies using median prescription doses >13Gy.

### Limitations

Limitations include systematic bias, type (retrospective) of most included studies, inconsistencies of outcome and treatment parameter reporting, and the differences between centers in patient selection and treatment patterns. Moreover, annual hemorrhage rates were not calculated consistently with the same methodology and data of patients with genetic mutations were not readily available. Finally, the radiosurgical techniques and devices used varied, with continuous evolution of radiosurgical technology and imaging protocols.

## Conclusions

SRS is effective in reducing hemorrhage rates for deep-seated ICMs. The risk of symptomatic radiation injury is low and generally acceptable. Given the high risk of surgical morbidity, SRS is a reasonable treatment for patients with deep-seated ICMs with a history of a prior, symptomatic hemorrhage.

## Supplementary information

ESM 1 (DOCX 1297 kb)

## Data Availability

No datasets were generated or analysed during the current study.
