# Supplementary material for: Stereotactic radiosurgery for intracranial cavernous malformations of the deep-seated locations: systematic review and meta-analysis
Source: Neurosurg Rev. 2024 Apr 24;47(1):186. doi: 10.1007/s10143-024-02434-9 (PMC11039535; doi:10.1007/s10143-024-02434-9)
Supplement: Supplementary file 1 — (DOCX 1297 kb) [file 10143_2024_2434_MOESM1_ESM.docx]

**Stereotactic Radiosurgery for Intracranial Cavernous Malformations of the Deep-seated Locations: Systematic review and Meta-analysis.
Running title:** SRS for Deep-seated ICM.
 **Supplemental materials:**
eTable 1: Databases & Search Strategy (From inception to January 9, 2024).
eTable 2: Summary of involved studies.
eFigure 1: Risk of bias using ROBINs-I, traffic (A) and summary (B) plots.
eFigure 2: Forest plots of annual hemorrhage rate comparing pre-SRS and total post-SRS for brainstem alone.
eFigure 3: Contour-enhanced funnel plot for pre-SRS vs post-SRS annual hemorrhagic rate.
eFigure 4: Forest plots of annual hemorrhage rate comparing pre-SRS and first 2 years post-SRS for brainstem alone.
eFigure 5: Contour-enhanced funnel plot for pre-SRS vs first 2 years post-SRS annual hemorrhagic rate.
eFigure 6: Forest plots of annual hemorrhage rate comparing pre-SRS and after 2 years post-SRS for brainstem alone.
eFigure 7: Contour-enhanced funnel plot for pre-SRS vs after 2 years post-SRS annual hemorrhagic rate.
eFigure 8: Forest plots of annual hemorrhage rate comparing first 2 years and after 2 years post-SRS for brainstem alone.
eFigure 9: Contour-enhanced funnel plot for first 2 years post-SRS vs after 2 years post-SRS annual hemorrhagic rate.
eFigure 10: Forest plot for symptomatic radiation-induced changes.
eFigure 11: Subgroup analysis forest plot for symptomatic radiation-induced changes according to prescription dose.
eFigure 12: Forest plot for permanent symptomatic radiation-induced changes.

eTable 1: Databases & Search Strategy (From inception to January 9, 2024).

| Database | Search strategy (Inception - January 9 2024) | Number of studies |
| --- | --- | --- |
| **Pubmed** | (Intracranial OR Cerebral* OR Brain) AND ("cavernous malformation*" OR "CCM" OR "cavernoma" OR "cavernous hemangioma" OR "Capillary Malformation*" OR "angiomatous malformation*" OR "cavernous angioma*" OR "cavernous angioma" OR "cavernous hemangioma" OR "cavernous haemangioma" OR "cavernous sinus hemangioma" OR "occult vascular malformations" OR "venous malformation") AND ("gamma*" OR "linear accelerator" OR "LINAC" OR "Cyberknife" OR "stereotactic radiosurgery" OR "SRS" OR "Stereotactic" OR "Proton" OR "particle" OR "charged particle" OR "hadron" OR "heavy ion") | 467 |
| **Scopus** | ( TITLE-ABS-KEY ( intracranial OR cerebral* OR brain ) AND TITLE-ABS-KEY ( "cavernous malformation*" OR "CCM" OR "cavernoma" OR "cavernous hemangioma" OR "Capillary Malformation*" OR "angiomatous malformation*" OR "cavernous angioma*" OR "cavernous angioma" OR "cavernous hemangioma" OR "cavernous haemangioma" OR "cavernous sinus hemangioma" OR "occult vascular malformations" OR "venous malformation" ) AND TITLE-ABS-KEY ( "gamma*" OR "linear accelerator" OR "LINAC" OR "Cyberknife" OR "stereotactic radiosurgery" OR "SRS" OR "Stereotactic" OR "Proton" OR "particle" OR "charged particle" OR "hadron" OR "heavy ion" ) ) | 716 |
| **WOS** | (TS=(Intracranial OR Cerebral* OR Brain) AND TS=("cavernous malformation*" OR "CCM" OR "cavernoma" OR "cavernous hemangioma" OR "Capillary Malformation*" OR "angiomatous malformation*" OR "cavernous angioma*" OR "cavernous angioma" OR "cavernous hemangioma" OR "cavernous haemangioma" OR “cavernous sinus hemangioma” OR "occult vascular malformations" OR "venous malformation")) AND TS=("gamma*" OR "linear accelerator" OR "LINAC" OR "Cyberknife" OR "stereotactic radiosurgery" OR "SRS" OR "Stereotactic" OR "Proton" OR "particle" OR "charged particle" OR "hadron" OR "heavy ion") | 436 |
| **Embase** | (intracranial OR cerebral* OR 'brain'/exp OR brain) AND ('cavernous malformation*' OR 'ccm' OR 'cavernoma'/exp OR 'cavernoma' OR 'capillary malformation*' OR 'angiomatous malformation*' OR 'cavernous angioma*' OR 'cavernous angioma'/exp OR 'cavernous angioma' OR 'cavernous hemangioma'/exp OR 'cavernous hemangioma' OR 'cavernous haemangioma'/exp OR 'cavernous haemangioma' OR 'cavernous sinus hemangioma'/exp OR 'cavernous sinus hemangioma' OR 'occult vascular malformations' OR 'venous malformation'/exp OR 'venous malformation') AND ('gamma*' OR 'linear accelerator'/exp OR 'linear accelerator' OR 'linac'/exp OR 'linac' OR 'cyberknife'/exp OR 'cyberknife' OR 'stereotactic radiosurgery'/exp OR 'stereotactic radiosurgery' OR 'srs' OR 'stereotactic' OR 'proton'/exp OR 'proton' OR 'particle' OR 'charged particle' OR 'hadron'/exp OR 'hadron' OR 'heavy ion'/exp OR 'heavy ion') | 846 |

**eTable 2:** Summary of involved studies.

| **Study information** | | | | | **Patient information** | | | | **CCMs Information** | | | | **Treatment Information** | | | | |
| --- | --- | --- | --- | --- | --- | --- | --- | --- | --- | --- | --- | --- | --- | --- | --- | --- | --- |
| **Author** | **Country** | **Institution (s) name** | **Study  design** | **Year of treatment** | **Patient (Lesion), n** | **Sex (M:F)** | **mean Age (years)** | **mean Follow up (months)** | **Location** | **Hemorrhages**  **Patients /CCMs (single/multiple)** | **Volume (cm^3^), mean** | **mean Max diameter (cm)** | **Previous Surgery** | **Previous SRS** | **SRS Parameters** | | |
|  |  |  |  |  |  |  |  |  |  |  |  |  |  |  | **SRS modality** | **Margin dose (Gy, mean)** | **Isodose line (%, mean)** |
| Lee CC 2012 | Taiwan | Taipei Veterans General Hospital | R, S | 1993 - 2010 | 49 (50) | 20:29 | 37.8 (7.4–73.9) | 40.6 | Brainstem = 50 | 49/49 (0/49) | 3.2  (0.1–14.6) | NA | NA | 0 | GKRS | 11.0  (9–15) | 60  (50–85) |
| Fuetsch 2012 | Germany | University of Cologne | R, S | 1992 - 2008 | 12 (12) | 6: 6 | 41* (19–71) | 85.2* (24 – 201.6) | Brainstem = 12 | 12/12 (3/9) | 1.6  (0.4–4.3) | NA | NA | NA | LINAC | 13.9* (11–18) | NA |
| Park 2013 | Korea | Kyungpook National University Hospital | R, S | 2005 - 2010 | 20 (22) | 11:9 | 41.1, 39.5* (24-69) | 38.9, 32* (12−82) | Brainstem = 22 | 20/22 (10/12) | 1.27, 0.56* (0.04-11.1) | NA | 1 | 0 | GKRS | 13*  (10 -17) | 50 |
| Lee SH 2014 | South Korea | Kyung Hee University Hospital | R, S | 1992 - 2011 | 49 (49) | 29:20 | 43.00 (13.98) | 40.8* | Brainstem = 49 | 49/49 (31/18) | 0.74* (0.03–8.37) | NA | 0 | 0 | GKRS | 13.1  (9-16.8) | 62.14  (50-80) |
| Frischer 2014 | Austria | Medical University of Vienna | CC, S | 1987–2011 | 38 (38) | 19:19 | 43.7* (19.3–76.8 ) | 62.4* | T/MB = 13 Pons = 23 Cerebellar peduncles = 2 | (NA/6) | 0.3*  (0.1–2.5) | NA | NA | NA | GKRS | 12*  (6–16) | 50*  (45–80) |
| Kim 2014 | South Korea | Samsung Medical Center | R, S | 1997 - 2012 | 39 (39) | 16:23 | 41.5  (18 - 64) | 49.2 | Brainstem = 39 | 39/39 (34/5) | 1.0953  (0.0315– 5.4) | NA | partial, 4 | NA | GKRS | 13*  (11–18) | 50* |
| Aboukais 2016 | France | Lille University Hospital | R, S | 2007 - 2012 | 19(19) | 10:9 | 36.7 (22–57) | 51.2  (24–77)  44.4* | Brainstem =19 | 19/19 (4/15) | 0.282 (0.048–0.813) | NA | NA | NA | GKRS | 14.8 (11-15) | NA |
| Park 2018 | Korea | Seoul National University Hospital | R, S | 1998 - 2011 | 45 (45) | 14:31 | 36.6  (3 - 67) | 111.72 (61.2 - 232.8) | Brainstem = 45 | 45/45 (26/19) | 1.88  (0.1-13.5) | NA | 3 | NA | GKRS | 13  (9 -16) | 50  (50-80) |
| Jacobs 2019 | USA | University of Pittsburgh | R, S | 1988 - 2016 | 76 (76) | 40:36 | 41.6  (5–79) | 72* | Brain stem = 76 | 76/76(7/69) | 0.66* (0.05–6.8) | NA | partial, 14 | NA | GKRS | 15.0* (12–20) | 52  (40-90) |
| Kefeli 2019 | Turkey | Acıbadem Kozyatagi Hospital | R, S | 2007 - 2015 | 82 (82) | 47: 35 | 41.5* (6-69) | 50 (13-113) | Brainstem = 82 | 82/82 (68/14) | 0.53, 0.3*  (0.1-3.6) | NA | 8 | NA | GKRS | 11.79, 12*  (10-13) | 50 |
| Nagy 2019 | UK | National Institute of Clinical Neurosciences, and  Thornbury Radiosurgery Centre | R, MC | 1995 - 2014 | 210 (210) | NA | 43*  (2–78) | 66* (12 – 240) | BG/thalamus = 55  Brainstem =155 | 210/210 (127/83) | BG/T 0.537* (0.0035–5.2)  Brainstem 0.240* (0.010–8.4) | NA | 21 | NA | GKRS | 12–13 | 50 |
| Hu 2021 | China | West China Hospital | R, S | 2009 - 2018 | 53 (55) | 25:28 | 43.1 (7.3–69.4) | 52.1 (6.2–104.3) | BG/thalamus = 54 Brainstem = 1 | 53/53 (29/24) | 1.77 (0.1–6.32) | NA | 2 | 0 | GKRS | 13.2 (12–16) | 50  (45-60) |
| Singh 2023 | International ^ | 11 centers^ | R, MC | 2001 - 2021 | 73 (73) | 37:36 | 43.5* (4.4–79.5) | 3 (0.55–18.44) | BG/thalamus = 73 | 59/59 (0-2) | 0.9* (0.07–10.1) | NA | NA | 0 | GKRS | 12* (10–20) | 50*  (20–90) |
| Yao 2023 | China | Beijing Tiantan Hospital | P, S | 2006 - 2015 | 85 (85) | 50:35 | 39.6, 41.0* (10–62) | 108.7, 121.9* (7.0–201.1) | BG/thalamus = 85 | 85/85 (57/28) | 1.534, 1.292* (0.050–7.342) | NA | 0 | 0 | GKRS | 14.7, 15*  (10-18) | 51.3, 50* (42-65) |

^ 11 centers (University of Virginia, Koc University, Nasser institute Hospital, Rúber Internacional Hospital, Université de Sherbrooke, PGIMER, IRCCS Istituto Clinico Humanitas, CEDIMAT, The Jewish Hospital - Mercy Health, NYU Langone Health, University of Alberta) at United States, Turkey, Egypt, Spain, Czech Republic, Canada, India, Italy, Dominican Republic.

* Reported as median.

BG, Basal ganglia; T, Thalamus; M, Midbrain; R, Retrospective; P, Prospective; S, Single-center; MC; Multicenter; GKRS, Gamma knife radiosurgery; LINAC, Linear accelerator.


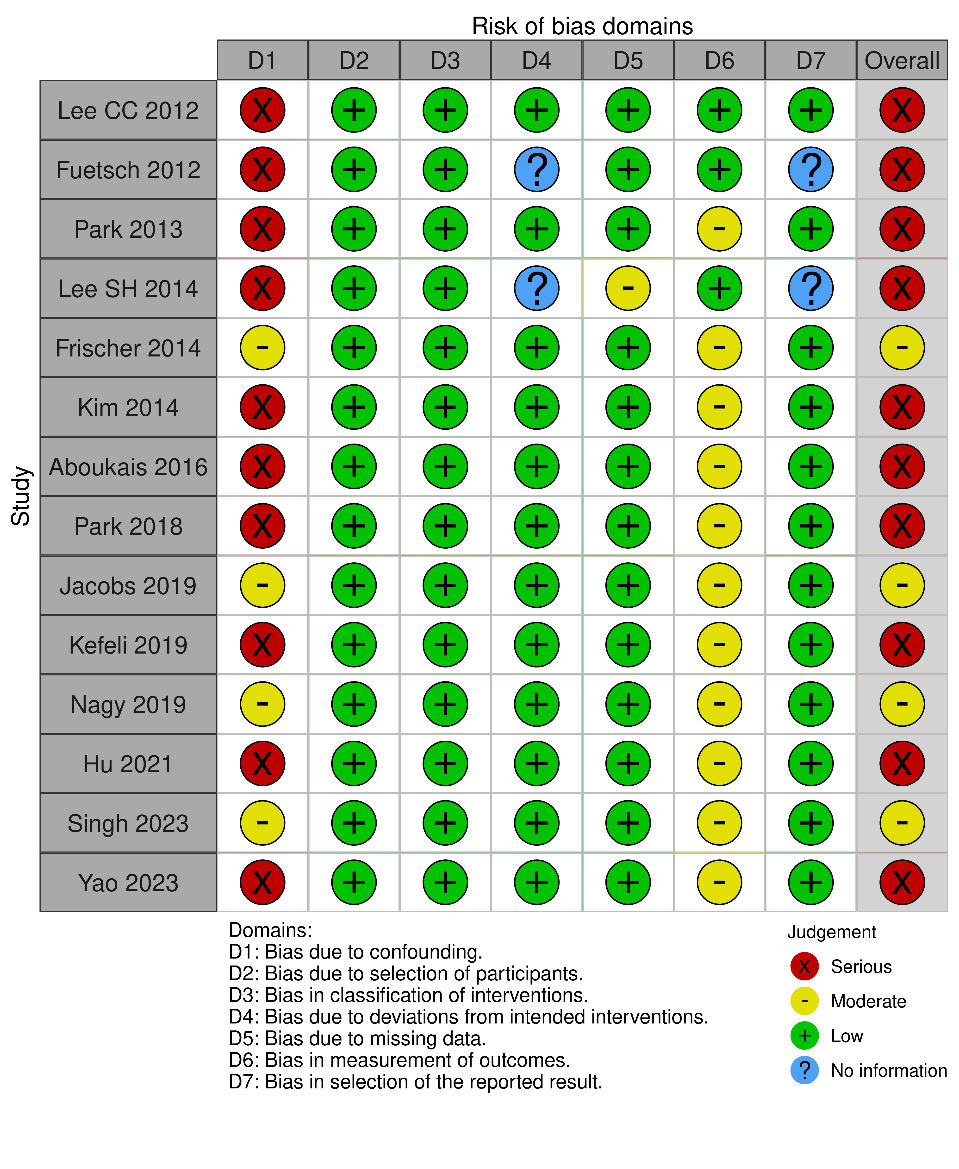


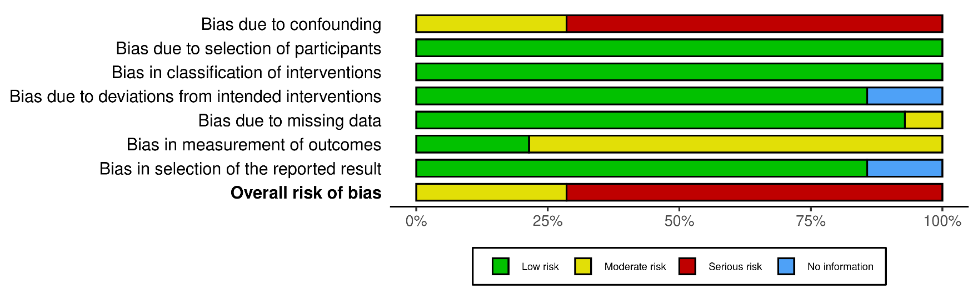

eFigure 1: Risk of bias using ROBINs-I, traffic (A) and summary (B) plots.


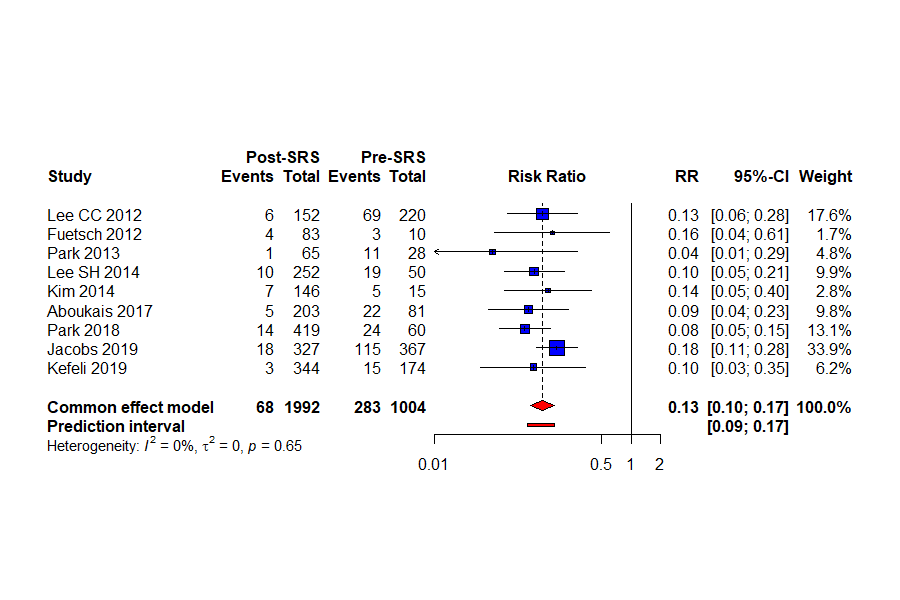

eFigure 2: Forest plots of annual hemorrhage rate comparing pre-SRS and total post-SRS for brainstem alone.

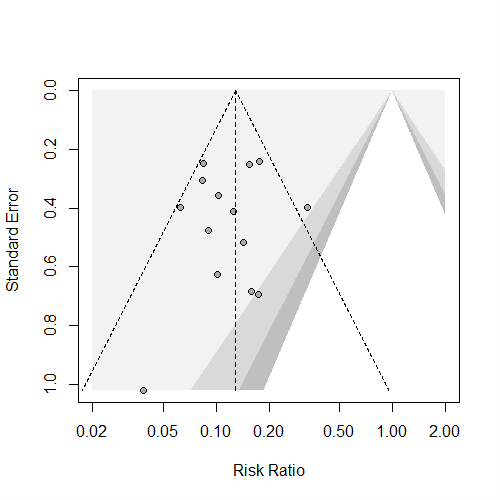

eFigure 3: Contour-enhanced funnel plot for pre-SRS vs post-SRS annual hemorrhagic rate.


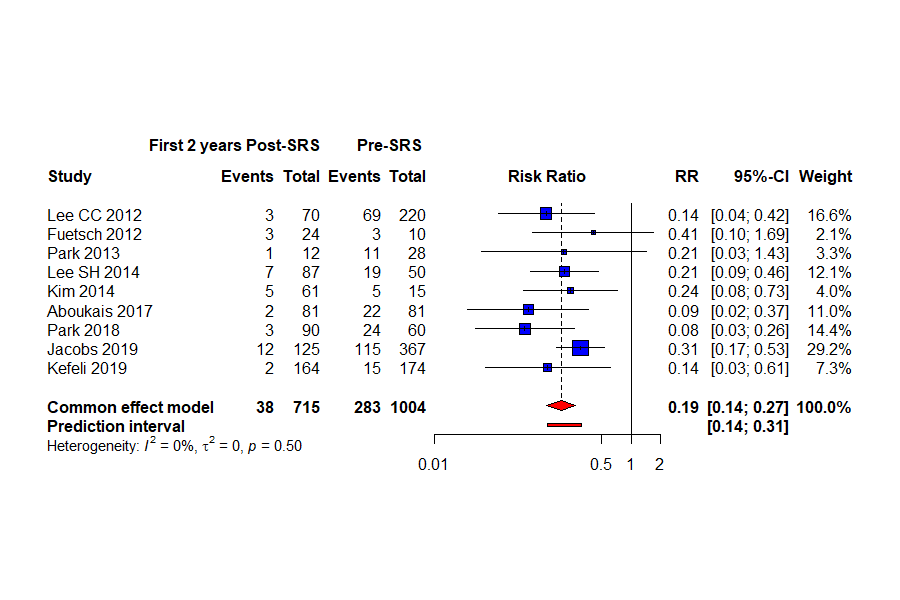

eFigure 4: Forest plots of annual hemorrhage rate comparing pre-SRS and first 2 years post-SRS for brainstem alone.


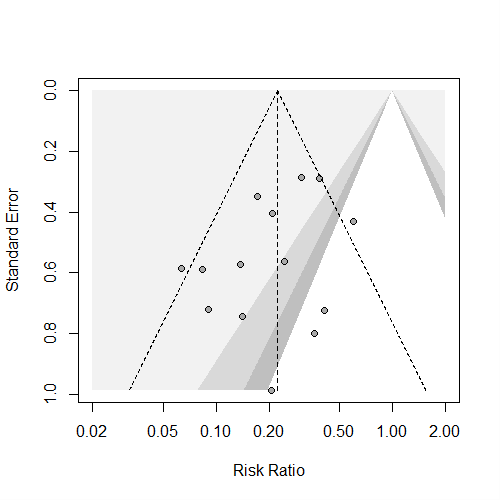


eFigure 5: Contour-enhanced funnel plot for pre-SRS vs first 2 years post-SRS annual hemorrhagic rate.


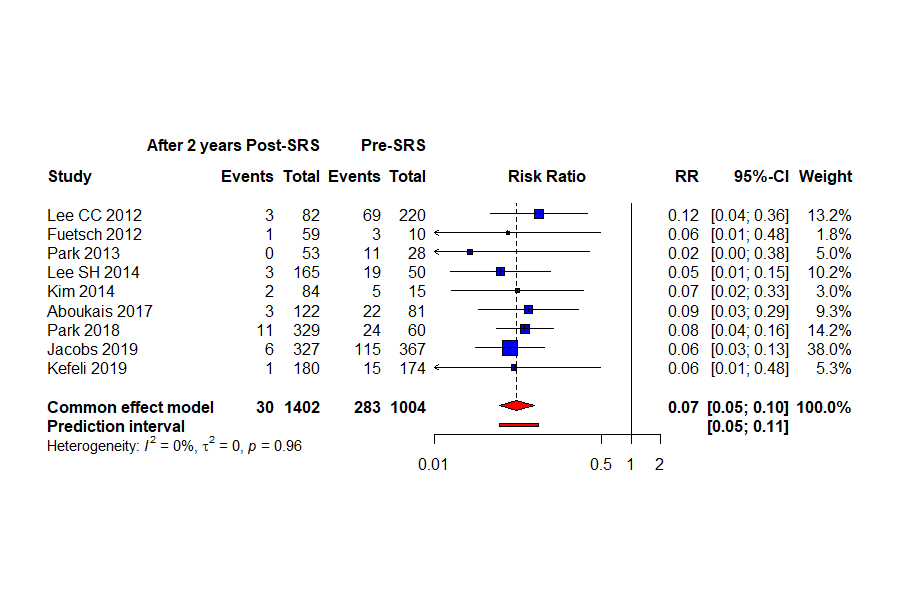

eFigure 6: Forest plots of annual hemorrhage rate comparing pre-SRS and after 2 years post-SRS for brainstem alone.

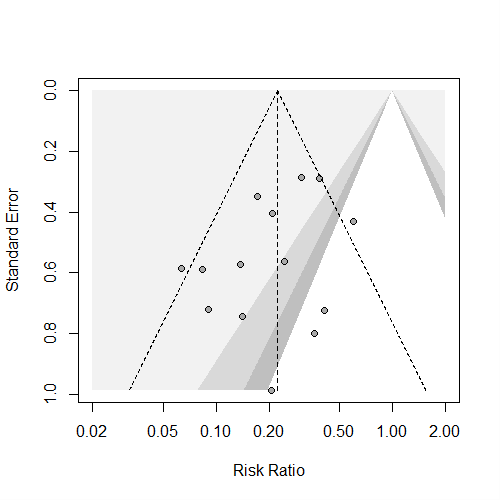

eFigure 7: Contour-enhanced funnel plot for pre-SRS vs after 2 years post-SRS annual hemorrhagic rate.

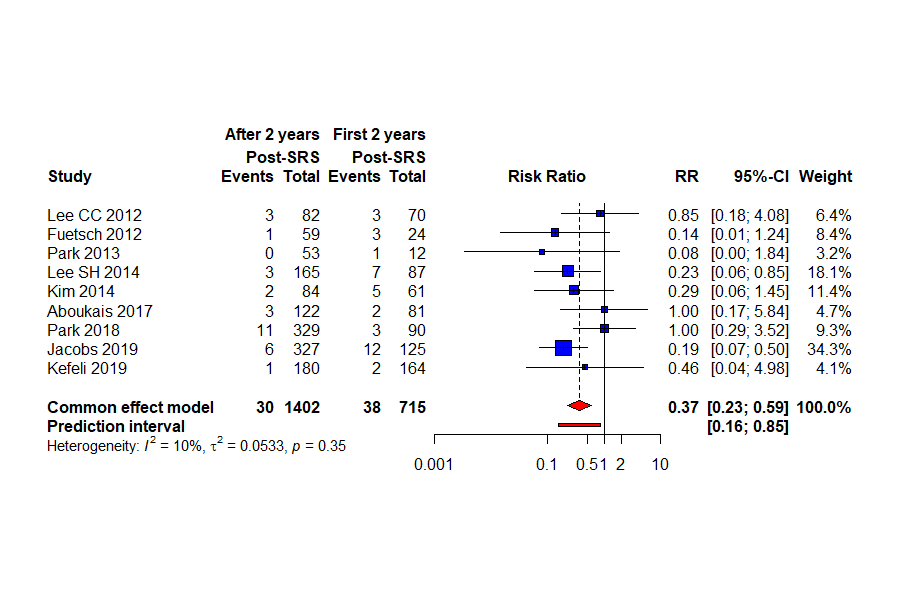

eFigure 8: Forest plots of annual hemorrhage rate comparing first 2 years and after 2 years post-SRS for brainstem alone.


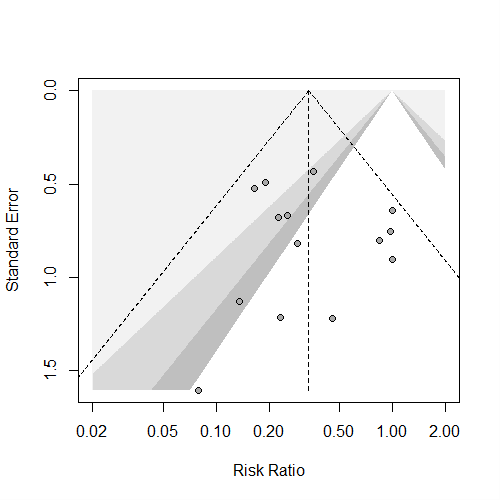

eFigure 9: Contour-enhanced funnel plot for first 2 years post-SRS vs after 2 years post-SRS annual hemorrhagic rate.

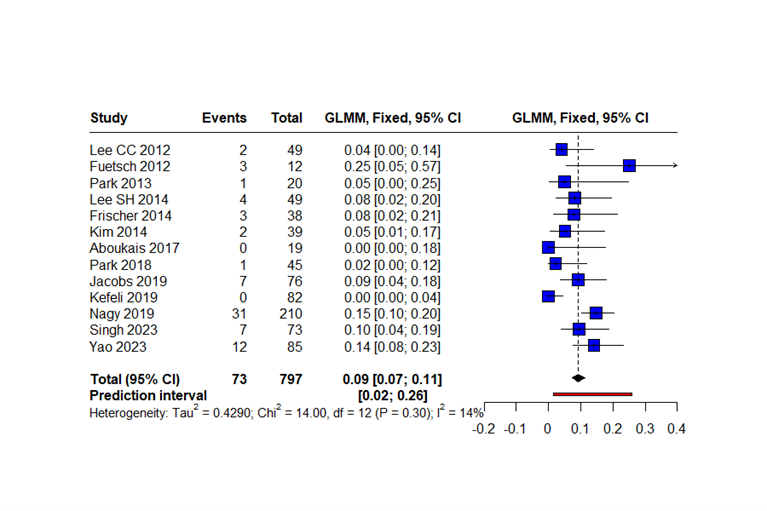

eFigure 10: Forest plot for symptomatic radiation-induced changes.


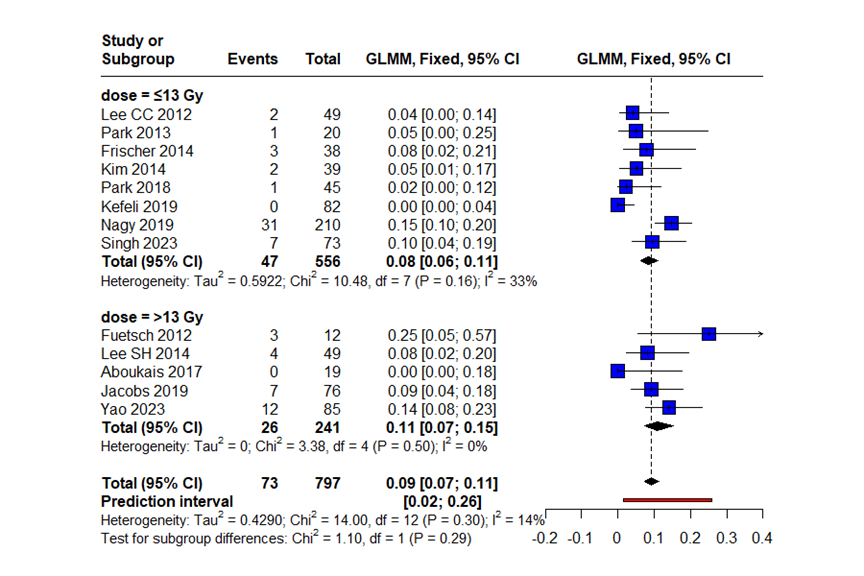

eFigure 11: Subgroup analysis forest plot for symptomatic radiation-induced changes according to prescription dose.

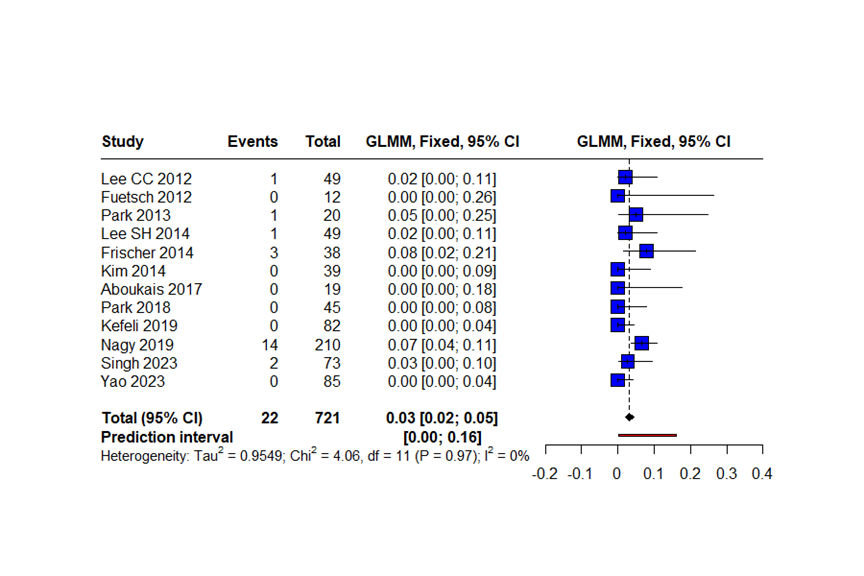


eFigure 12: Forest plot for permanent symptomatic radiation-induced changes.
